# Screening of *Candida* spp. in wastewater in Brazil during COVID-19 pandemic: workflow for monitoring fungal pathogens

**DOI:** 10.1186/s12896-024-00868-z

**Published:** 2024-06-22

**Authors:** Danielly Corrêa-Moreira, Gisela Lara da Costa, Reginaldo Gonçalves de Lima Neto, Tatiana Pinto, Bruna Salomão, Tulio Machado Fumian, Camille Ferreira Mannarino, Tatiana Prado, Marize Pereira Miagostovich, Lívia de Souza Ramos, André Luis Souza dos Santos, Manoel Marques Evangelista Oliveira

**Affiliations:** 1 Laboratory of Taxonomy, Biochemistry and Bioprospecting of Fungi, Oswaldo Cruz Institute, Oswaldo Cruz Foundation, Rio de Janeiro, 21040-360 Brazil; 2 Federal University of Pernambuco, UFPE, Pernambuco, 50670901 Brazil; 3 Medical Microbiology Department, Paulo de Goés Institute of Microbiology, Federal University of Rio de Janeiro, Rio de Janeiro, 21941-909 Brazil; 4 Laboratory of Microbiology, Federal Hospital of Andaraí, Rio de Janeiro, 20541-173 Brazil; 5 Laboratory of Comparative and Environmental Virology, Oswaldo Cruz Institute, Oswaldo Cruz Foundation, Rio de Janeiro, 21040-360 Brazil; 6 Laboratory of Respiratory, Exanthematic, Enteric viruses and Viral Emergencies, Oswaldo Cruz Institute, Oswaldo Cruz Foundation, Rio de Janeiro, 21040-360 Brazil; 7 Laboratory for Advanced Studies of Emerging and Resistant Microorganisms, General Microbiology Department, Institute of Microbiology Paulo de Góes, Federal University of Rio de Janeiro, Rio de Janeiro, 21941-909 Brazil

**Keywords:** *Candida* species, Emergent fungi, Polyphasic taxonomy, Fungal virulence factors, COVID-19

## Abstract

Fungal diseases are often linked to poverty, which is associated with poor hygiene and sanitation conditions that have been severely worsened by the COVID-19 pandemic. Moreover, COVID-19 patients are treated with Dexamethasone, a corticosteroid that promotes an immunosuppressive profile, making patients more susceptible to opportunistic fungal infections, such as those caused by *Candida* species. In this study, we analyzed the prevalence of *Candida* yeasts in wastewater samples collected to track viral genetic material during the COVID-19 pandemic and identified the yeasts using polyphasic taxonomy. Furthermore, we investigated the production of biofilm and hydrolytic enzymes, which are known virulence factors. Our findings revealed that all *Candida* species could form biofilms and exhibited moderate hydrolytic enzyme activity. We also proposed a workflow for monitoring wastewater using Colony PCR instead of conventional PCR, as this technique is fast, cost-effective, and reliable. This approach enhances the accurate taxonomic identification of yeasts in environmental samples, contributing to environmental monitoring as part of the One Health approach, which preconizes the monitoring of possible emergent pathogenic microorganisms, including fungi.

## Introduction

The rise in global temperatures due to greenhouse gas emissions enhances the pathogenic potential of fungi by allowing adaptation to higher temperatures, making them capable of surviving at mammalian body temperatures [1]. Fungal diseases, often linked to precarious hygiene and sanitation conditions, are exacerbated by pandemics. In December 2019, cases of severe respiratory distress of unknown cause emerged in Wuhan, China. The identified cause, SARS-CoV-2, quickly spread within China and beyond [2–5]. In January 2020, WHO declared the COVID-19 outbreak a Public Health Emergency of International Concern, particularly threatening countries with weak health systems [6]. By March 11, 2020, COVID-19 was declared a pandemic. Brazil confirmed its first case on February 26, 2020, in São Paulo, with Rio de Janeiro following a week later [7].

COVID-19 can cause severe respiratory issues and immunosuppression, marked by decreased TCD4+/TCD8 + cells and increased pro-inflammatory cytokines [8, 9]. ICU hospitalization for COVID-19, involving intubation, mechanical ventilation, and corticosteroid therapy, heightens the risk of invasive fungal infections (IFI) [9, 10]. The use of dexamethasone, a common COVID-19 treatment, further suppresses immune responses by affecting IL-2 synthesis, crucial for TCD4 + lymphocyte proliferation [11].

The One Health framework integrates the public health, veterinary, and environmental sectors. It is particularly relevant for Mycology since it concerns controlling zoonotic diseases, managing pollution, and combating antimicrobial resistance. In the context of the millions of species in the Fungi Kingdom, this approach discusses the positive and negative impact of these organisms on plants, human and animal health, and the role of the environment as an influencing factor in the emergence and re-emergence of fungi potentially pathogenic species [12]. Hyde [13], states that the Fungi Kingdom is the largest in terms of species diversity, with between 2.2 and 3.8 million estimated species, and of these, only 150,000 are described. The author highlights that fungi and species tracking, through different methods and strategies, are essential for the functioning of the ecosystem.

Among the species of fungi, yeasts, and mainly the genus *Candida* stand out, which includes around 150 species, some of them living in symbiosis in the microbiota of the reproductive and gastrointestinal mucosa of 50–70% of healthy individuals. These fungi have an opportunistic profile, causing infections in immunosuppressed hosts, mainly neutropenic patients, as well as in those undergoing treatment with broad-spectrum antimicrobials, parenteral nutrition, and invasive examinations. These conditions make *Candida* spp. important causative agents of candidiasis, which can be superficial or invasive [9, 14, 15].

*Candida albicans-related* infections represent around 80% of the more frequent infections caused by these species. However, non-*albicans Candida* species (*Candida glabrata*, *Candida tropicalis*, *Candida krusei*, *Candida dubliniensis*) are becoming more frequent [15]. Moreover, *Candida auris* has emerged as multi-drug resistant yeast, responsible for the major issues regarding patient treatment and surface disinfection in hospitals [16, 17].

In this sense, considering that climate change also favors the emergence of new virulent fungal lineages and long-distance spore dispersal, the prediction of where and when emergent fungal pathogens will appear, and establishing surveillance protocols such as the early detection of these pathogens, is essential. Diagnosing invasive candidiasis depends on culture methods and applied phenotypic and genotypic tools [9], which promote the accurate and early detection and identification of emergency fungal agents. Furthermore, preparing health system institutions and organizations to assist in choosing the best therapeutic regimen and diagnostic strategy, may define the outcome of the disease.

Based on these questions, the present study seeks to carry out a screening of yeasts and Sars-CoV-2 in wastewater, observing their biological diversity and pathogenicity profiles. Furthermore, we propose a workflow for quick and accurate identification of environmental samples, using polyphasic taxonomy.

## Methods

### Study area and sewage sampling

**Fig. 1:**
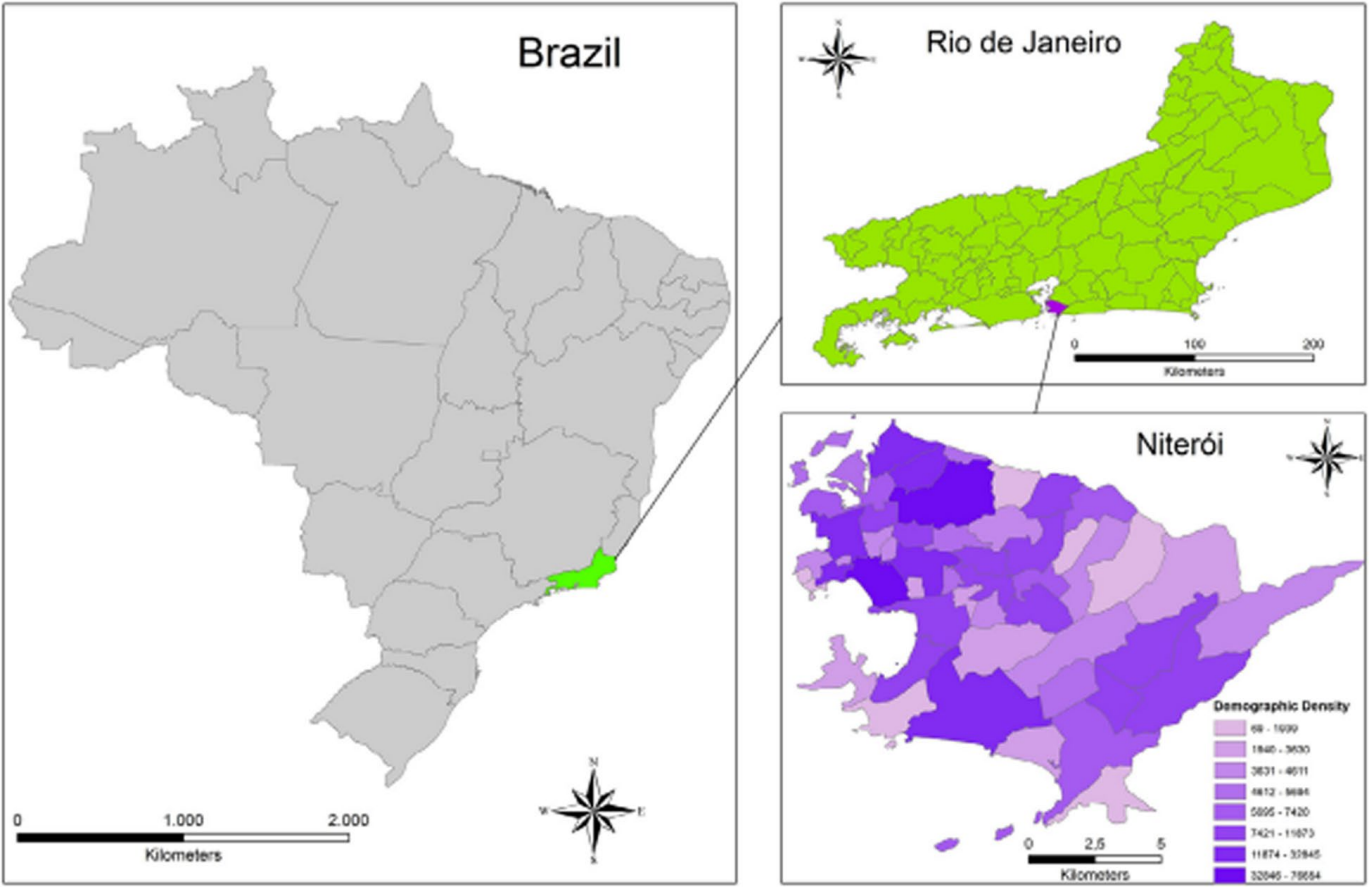
Location of Niterói on maps of Brazil and Rio de Janeiro State, indicating range of demographic density. B Sampling points distributed in Niterói municipality– location map (Reproduced from [7])

Sewage samples were monitored weekly and collected during the first wave of SARS-COV-2, in the municipality of Niteroi, state of Rio de Janeiro, Brazil (Fig. 1), according to Prado et al. [7]. 10 h-composite samples were stored in sterile polypropylene bottles and transported at 4 °C to the Laboratory of Comparative and Environmental Virology of the Oswaldo Cruz Foundation, where they have been pasteurized at 60 °C for 90 min to inactivate the coronaviruses. As described by Prado et al. [7], to detect the virus, briefly, 42 mL of sewage samples were centrifuged, and after supernatant discharge, the pellet was re-suspended in 4 mL of 0.25 N glycine buffer (pH 9.5), incubated, and mixed by vortex each 5 min. The solution was then neutralized and clarified by centrifugation. Supernatant samples were centrifuged, viral particles were re-suspended in PBS (pH 7.2) and processed immediately for nucleic acid extraction or stored at − 80 °C until use.

Afterward, yeasts were isolated from the pre-established point of 45 mL of each sample and centrifuged at 4000 rpm for 5 min at the Laboratory of Taxonomy, Biochemistry and Bioprospecting of Fungi, Oswaldo Cruz Foundation. The supernatant was removed, and the pellet was resuspended in 250 µl of 0.9% saline solution.

### Quantification of yeasts colony-forming unit (CFU)

Briefly, 20 µl of the solution described in heading 2.1 were deposited on two Petri dishes containing Sabouraud Dextrose Agar (SDA) medium (Difco, Becton-Dickinson and Company, USA), plus 400 mg/L of chloramphenicol and 25 mg/L of gentamicin and incubated at 35 °C for 5 days (Workflow in supplementary material 1). After, the colonies were quantified in UFC and each representative of them was transferred to test tubes containing SDA, for performed macroscopic description of the colony (adapted from [18]).

### Phenotypic characterization

After the growth of the selected colonies in slant agar tubes containing SDA, at 35 °C for 24 h, 1 µL of the yeast suspension in 0.9% saline solution, equivalent to standard n° 1 of the McFarland scale was seeded in Petri dishes, containing the chromogenic medium CHROMagar® *Candida* (Difco, Becton-Dickinson and Company, USA). The plates were incubated at 37 °C for 48 h to assess their purity and the results were interpreted based on manufacturer’s guidelines, as follows: *C. albicans* - colonies light green to medium green, *C. tropicalis* - blue-gray, bluish-gray to blue-green or metallic blue colonies with or without violet halos in the middle, *C. krusei* - large flat colonies, light pink to light red with a whitish border, and nonspecific nuclei, other species. CHROMagar Candida Plus™ (CHROMagar, France) was used to evaluate the presence of *C. auris* species in the samples since light-blue colonies and a blue halo are described by the manufacturer as a suggestion of *C. auris*.

Additionally, to evaluate metabolic properties, sugar assimilation and enzymatic reactions were performed and analyzed by the VITEK 2 system (bioMerieux, France), using a YST card according to the manufacturer’s guidelines.

### Molecular identification

#### MALDI-TOF MS

To identify the samples at the species level, MALDI-TOF MS was performed, according to Pinto et al. [19]. Briefly, yeast cells suspension at 10^6^ were transferred from the culture plate to a tube containing 20 µl of 70% formic acid in water (v/v) and 1 µl of supernatant of each sample (in triplicate) was mixed with 10 µl of acetonitrile. Then, the sample was placed onto the MALDI-TOF MS stainless plate (Bruker Daltonics, Germany) and covered with 1 µl matrix solution α-cyano-4-hydroxycinnamic acid (CHCA, Fluka, Buchs, Switzerland). After drying, the spectra acquisition was done using the Bruker database to identify the isolate at the species level.

#### Colony PCR

Yeats were grown in SDA plates at 30 °C for 48 h. To perform colony PCR, a small portion of an isolated colony was picked using a micropipette tip and added to the PCR tubes as the DNA template. The cells were then heated in a microwave for 90 s and immediately placed on ice to prevent DNA degradation. Amplification was performed using 25ng of genomic DNA obtained in a 50-µl reaction volume, using 10 pmol of universal fungal primers ITS1 (CGTAGGTGAACCTGCGG) and ITS4 (TCCTCCGCTTATTGATATGC), according to Lindsley et al. [20]. The annealing temperature of the reactions was 58 °C, carried out in a 96-well thermocycler (Applied Biosystems by Thermofisher Scientific).

To purify the amplicons was used QIAquick® PCR Purification Kit (QIAGEN®) according to the manufacturer’s protocol [19]. The sequences obtained in the Sequencing Platform at Fundação Oswaldo Cruz - PDTIS/FIOCRUZ, Brazil, were edited using the CodonCode Aligner software and compared by BLAST (Basic Local Alignment Search Tool) with sequences available from NCBI / GenBank. Neighbor-joining algorithm of Saitou and Nei [21], with 1000 replicate bootstraps, was used to perform the phylogenetic analysis.

### Production of hydrolytic enzymes

To evaluate the production of hydrolytic enzymes followed the protocols of Rüchel et al. [22] and Price et al. [23], with modifications. Briefly, to determine aspartic protease activity, 1.17% yeast carbon base medium supplemented with 1% bovine serum albumin (BSA) was used. Phospholipase activity was determined using an egg yolk agar plate (Sabouraud dextrose agar supplemented with 1 M NaCl, 5 mM CaCl2, and 2% sterile egg yolk emulsion, pH 7.0). To evaluate the production of esterase, Tween agar plates were used (peptone, 10 g; NaCl, 5 g; CaCl2, 0.1 g; agar, 1.5%; Tween, 0.1%; pH 7.0 in 1000 mL of distilled water) according to Aktas et al. [24], with modifications. Finally, calcium phytate agar plates [glucose, 10 g; (NH4)2SO4, 0.5 g; KCl, 0.2 g; MgSO4.7H2O, 0.1 g; calcium phytate, 2 g; yeast extract, 0.5 g; MnSO4, 0.005 g; FeSO4, 0.005 g; agar, 15 g; pH 7.0 in 1000 mL of distilled water], to verify phytase activity [25].

After 48 h, 10 µL of fungal cell suspension were taken at a concentration of 1 × 10^7^ cells/mL, stained on the surface of each agar medium, and incubated at 37 °C for up to 7 days. The production of hydrolytic enzymes was expressed as Pz (a/b) value, where (a) corresponds to the colony diameter and (b) colony diameter plus the hydrolysis/precipitation zone [23]. The Pz value was scored in four categories: (a) Pz 1.0: no production; (b) Pz 0.999–0.700: weak producers; (c) Pz 0.699–0.400: good producers; and (d) Pz less than 0.399: excellent producers [23].

### Biofilm formation and biomass quantification

In 96-well plates, 200µL of the cell suspension in Sabouraud broth containing 10^6^ cells was incubated at 37 °C for 48 without shaking. Then, the supernatant was removed, and the wells were washed 3-times with phosphate-buffered saline (PBS). To quantify the biomass, the biofilms were fixed with 200 µL of 99% methanol for 15 min and the supernatant was discarded. After drying the plates, 200 µL of 0.4% crystal violet solution (Sigma-Aldrich, St Louis, MO, USA) was added to each well and the plates were incubated at room temperature for 20 min. Then, the plates were washed to remove excess dye and the biomass was decolorized with 200 µL of 33% acetic acid for 5 min. One hundred microliters of this suspension were transferred to a new 96-well plate and the absorbance was measured at 590 nm using a SpectraMax M3 microplate reader (Molecular Devices, Sunnyvale, CA, USA) [26].

## Results

### Sample collection and viral detection

Twelve different samples were isolated in the points of collection (ESG03, ESG04, ESG07, ESG12, ESG13, ESG15, ESG17, ESG19, ESG20, ESG21, ESG22, and ESG23) and, in five of them, was possible to detect SARS-CoV-2 and quantify the viral load – VL (GC/100mL): ESG03 – VL 4.57; ESG13 – VL 4.59; ESG15 – VL 3.85; ESG17 – VL3.85 an ESG20 – VL3.67.

It was also observed in two samples collected from the same source (ESG19 and ESG20 from point 9), only one was positive for Sars-Cov-2 (ESG 20).

### Fungal species identification

**Fig. 2:**
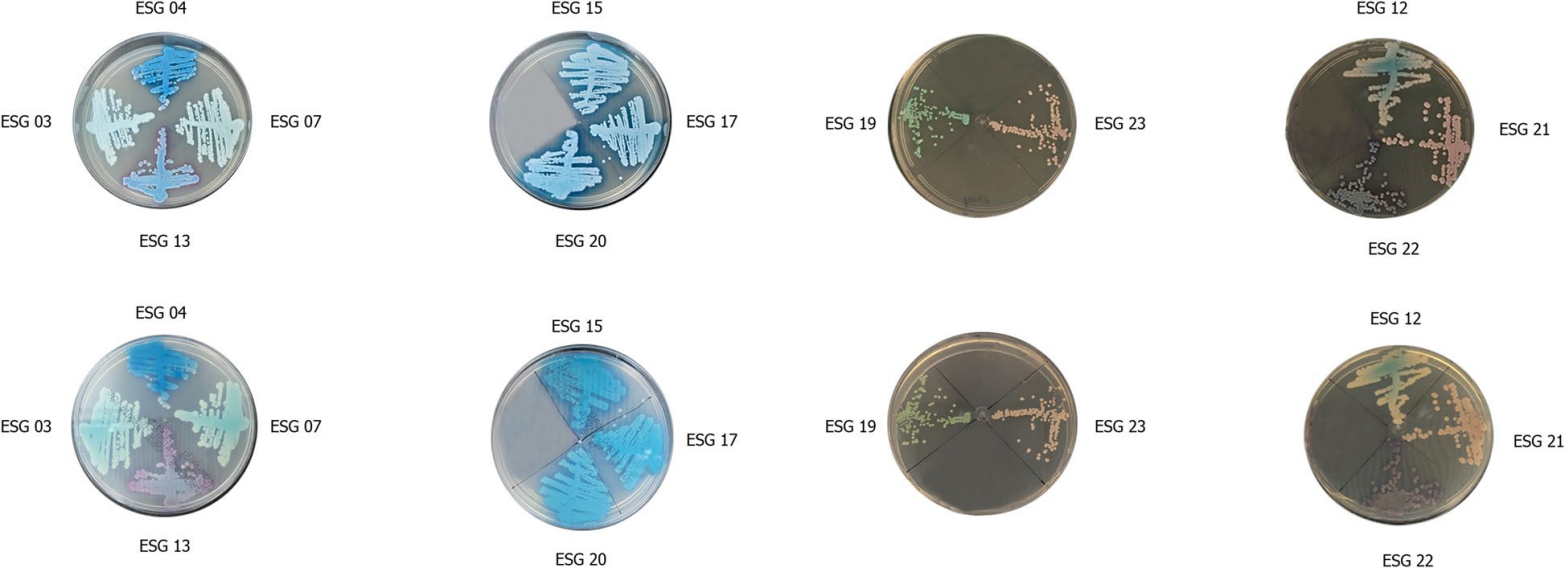
*Candida* species identification in Chromogenic medium ChromAgar *Candida*, after incubation at 37°C for 48 hours

Macroscopic aspects of the colonies on SDA and CHROMagar *Candida* medium revealed that all isolates are different species belonging to *Candida* genera (Fig. 2A). In the chromogenic medium, the isolates showed color variation and the identification did not conclude at the species level in most samples. *C. albicans* was suggested as a species only in one isolate (ESG 19). Then, the biochemical analysis of carbohydrate assimilation was performed by the Vitek 2 system and, except for the ESG 3 and ESG 13 isolates that were classified as undetermined (when the system identifies more than two possible species), and one sample (ESG 19) unidentified, the most frequent species were *C. parapsilosis* and *C. famata* (Fig. 2B). However, when performing molecular analysis by MALDI-ToF and ITS sequencing, the most frequently identified species was *C. palmioleophila* (58.33%). The identification of the samples by the different methods is summarized in Table 1.

**Table 1:** Sample identification by biochemical analysis (VITEK 2 system) and molecular methods

Sample	VITEK2	MALDI-TOF-MS	ITS Sequencing
ESG03	*C. albicans or C. famata or C. parapsilosis*	*C. palmioleophila*	*C. palmioleophila*
ESG04	*Kodamaea ohmeri*	*C. palmioleophila*	*C. palmioleophila*
ESG07	*C. parapsilosis*	*C. palmioleophila*	*C. palmioleophila*
ESG12	*C. guilliermondii*	*C. guilliermondii*	*C. guilliermondii*
ESG13	*C. tropicalis or C. parapsilosis*	*C. palmioleophila*	*C. palmioleophila*
ESG15	*C. famata*	*C. palmioleophila*	*C. palmioleophila*
ESG17	*C. parapsilosis*	*C. palmioleophila*	*C. palmioleophila*
ESG19	*----*	*C. albicans*	*C. albicans*
ESG20	*C. famata*	*C. palmioleophila*	*C. palmioleophila*
ESG21	*C. krusei*	*C. krusei*	*C. krusei*
ESG22	*C. tropicalis*	*C. tropicalis*	*C. tropicalis*
ESG23	*C. utilis*	*C. utilis*	*C. utilis*

--- not identified

### Hydrolytic enzyme and biofilm production

Regarding the production of Aspartic Protease and Calcium Phytate, five isolates (41,66%) were good producers and presented moderated activity with Pz values ranging from 0.67 to 0.51 and from 0.68 to 0.58, respectively. It was also observed that, except for two isolates (ESG 04 and PV 04), all the other samples were good Esterase producers (83,33% - Pz values 0.67–0.55). We highlight that ESG19 (*C. albicans*), ESG22 (*C. tropicalis*), and ESG23 (*C. utilis*) were the only isolates capable of producing three hydrolytic enzymes. On the other hand, none of the samples was able to produce Phospholipase (Table 2).

**Table 2:** Production of Hydrolytic enzymes by the isolates

Samples	AP	Phos	Est	CP
	Mean ± SD	Mean ± SD	Mean ± SD	Mean ± SD
ESG03 - *C. palmioleophila*	1.00 ± 0.00	1.00 ± 0.00	0.64 ± 0.03	1.00 ± 0.00
ESG04 - *C. palmioleophila*	0.53 ± 0.09	1.00 ± 0.00	1.00 ± 0.00	0.67 ± 0.07
ESG07 - *C. palmioleophila*	1.00 ± 0.00	1.00 ± 0.00	0.62 ± 0.02	1.00 ± 0.00
ESG12 - *C. guilliermondii*	1.00 ± 0.00	1.00 ± 0.00	0.67 ± 0.02	1.00 ± 0.00
ESG13 - *C. palmioleophila*	0.51 ± 0.02	1.00 ± 0.00	0.65 ± 0.02	0.65 ± 0.02
ESG15 - *C. palmioleophila*	1.00 ± 0.00	1.00 ± 0.00	0.57 ± 0.01	1.00 ± 0.00
ESG17 - *C. palmioleophila*	1.00 ± 0.00	1.00 ± 0.00	0.55 ± 0.02	1.00 ± 0.00
ESG19 - *C. albicans*	0.59 ± 0.01	1.00 ± 0.00	0.62 ± 0.02	0.58 ± 0.01
ESG20 - *C. palmioleophila*	1.00 ± 0.00	1.00 ± 0.00	0.61 ± 0.02	1.00 ± 0.00
ESG21 - *C. krusei*	0.71 ± 0.02	1.00 ± 0.00	1.00 ± 0.00	0.74 ± 0.01
ESG22 – *C. tropicalis*	0.63 ± 0.07	1.00 ± 0.00	0.62 ± 0.01	0.68 ± 0.03
ESG23 – *C. utilis*	0.67 ± 0.03	1.00 ± 0.00	0.64 ± 0.03	0.61 ± 0.02

*SD* Standard Deviation, *AP* Aspartic Protease, *Phos *Phospholipase, *Est *Esterase, *CP* Clacium Phytate

3.4 Biofilm formation

**Fig. 3:**
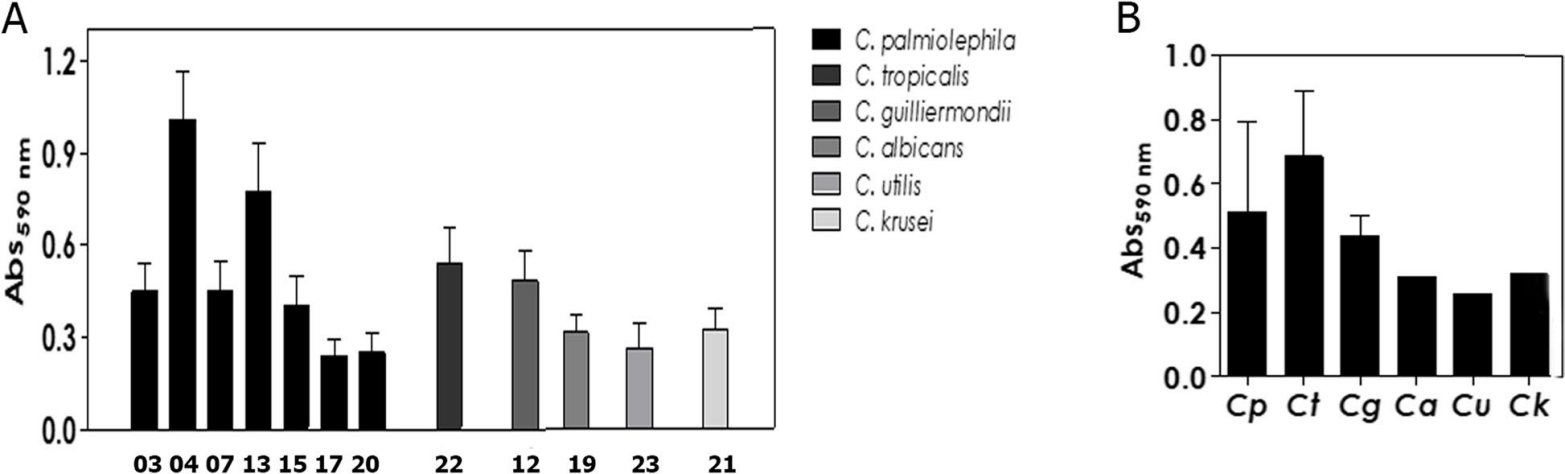
Biofilm formation by the *Candida* species on polystyrene surface and detection of fungal biomass by crystal violet incorporation in methanol-fixed biofilms at 590 nm The results were expressed as: **A** absorbance (ABS) and **B** mean per fungal species

Figure 3 shows the mean absorbance values of the biomass produced by the samples, after 48 h of incubation at 37 °C, ranging from 0.152 to 1.077. The ESG 04 and ESG 13, identified as *C. palmioleophila*, followed by ESG22, identified as *C. tropicalis*, showed the highest absorbance values. On the other hand, samples ESG 017 and ESG 20, both also identified as *C. palmioleophila*, presented the lowest absorbance values.

## Discussion

Herein, used for the first time the workflow that proposes a fast method for screening yeast species associated with the environmental samples of monitoring SARS-CoV-2 from wastewater samples obtained from different areas of Niterói municipality, state of Rio de Janeiro state, Brazil. According to the biosafety measures preconized by Wu et al. [27] and Prado et al. [7], the sewage samples containing viral particles are heat-inactivated, guaranteeing biosafety in the screening of fungal samples.

During the COVID-19 pandemic, several studies have shown that wastewater analysis is an important tool for monitoring the spread of SARS-CoV-2 in some regions [7, 28–30]. However, regarding the monitoring of fungal species, the reports of association of *Candida* species with sewage samples, especially regarding highly thermophilic species, are rare. We can cite the paper of Hautala et al. [31], previously to the pandemic, that relates the occurrence of *C. krusei* in two sewage samples, but the authors consider these samples an unlikely source of infection to the patients. The resistance of fungi to high temperatures is highlighted in the present work, not only because they have survived the use of high temperatures for the inactivation of viral particles, but mainly because this reflects the evolutionary adaptations of these microorganisms in response to climate change since physiological adaptations by fungal species to global warming have brought new fungal threats [32]. It concerns to significant challenges inherent to this group of organisms, which include their capacity for rapid evolution, the lack of vaccines, and, mainly, the increase in drug resistance [32].

In this work, 12 samples were collected, and from them, six *Candida* species were isolated. The most frequent species found was *C. palmioleophila*, a species associated with wild populations of *Spheniscus magellanicus* penguins [33], as a bioremediation agent for the degradation of artificial azo dyes, under saline conditions [34], and isolated from marine ecosystems, such as marshes and platform sediments continental [35]. Environmental interactions indicate a potentially pathogenic species, resistant to extreme temperatures and high osmotic levels, as observed by Lapeña et al. [36], who related thermotolerance of *C. utilis* induced by changes in light-dark cycles. This species, considered non-pathogenic, and largely used in the food industry, was cited by some authors as the causal agent of candidemia and in some cases, resistant to antifungal drugs [37–40].

It is important to mention that this resistance to inhospitable conditions is not exclusive to *C. palmioleophila*, but to other *Candida* species. Several authors have described the osmotic adaptive metabolism of *Candida albicans* complex [41–44]. *C. tropicalis*, a species related to candidemia in Pakistan, India, Thailand, and Algeria, and the second causal agent of candidemia in Brazil [45] is also described as an osmotolerant species [46]. Thus, we argue that the detection of *Candida* species in wastewater in Brazil is a future concern for public health conjectures since wastewater is profusely discharged, even without treatment, in different aquatic environmental waters, such as beaches and rivers.

As aforementioned, antifungal resistance constitutes a threat to public health, and one of the most effective measures to minimize it is the rapid and accurate identification of the etiological agent, thus avoiding the use of broad-spectrum antifungals or those whose action on a particular fungus is ineffective or non-existent.

In this sense, due to the presumptive nature of CHROMagar Candida medium, especially in the case of emerging species, also observed in Vitek-2 automated system since the scarcity of some species in our database, phenotypic identification presents limitations that can lead to mistaken identification. As an example, in Denmark, *C. palmioleophila* is frequently misidentified as other *Candida* species such as *C. famata* and *C. guilliermondii* by the approach of traditional methods [47–49]. In Italy, it was reported two cases of candidemia, due to *C. palmioleophila*, which was misidentified as *C. albicans* by using the Vitek2 system and CHROMagar *Candida* in the initial diagnosis [50, 51] compared phenotypical and molecular methods for *Candida* spp. identification and demonstrated that phenotypic methods were insufficient for correct identification. Additionally, most of the wrongly identified strains showed a resistant antifungal profile, such as *C. haemulonii*, *C. ciferri*, and *C. rugosa*, reinforcing the importance of correct identification. It is important to mention that our group published a study [52] using *C. palmioleophila* isolates that showed a characteristic MIC indicating resistance to fluconazole, corroborating the findings of the authors that point out that resistance to some azole derivatives among species of the Saccharomycotina subphylum, in which *Candida* species are inserted, is associated with multifactorial circumstances, such as indiscriminate exposure to azoles, patient profiles, geographic location of the species and genetic particularities [53, 54].

When comparing the species identification by the Vitek-2 system and molecular tools, it is possible to note a discrepancy in our results. While there was observed discordance of results between the morphological and biochemical analysis, the ITS sequencing and MALDI-ToF results converged 100% with each other. These results corroborate the findings of other authors, that reported biochemical identification as an inaccurate approach [49, 50]. It is also important to note that in our work, we reported the wrong identification of *Candida tropicalis*, *Candida parapsilosis*, and *Kodamaea ohmeri* by Vitek 2. This is important information for professionals who perform the mycological routine of yeast identification.

**Fig. 4:**
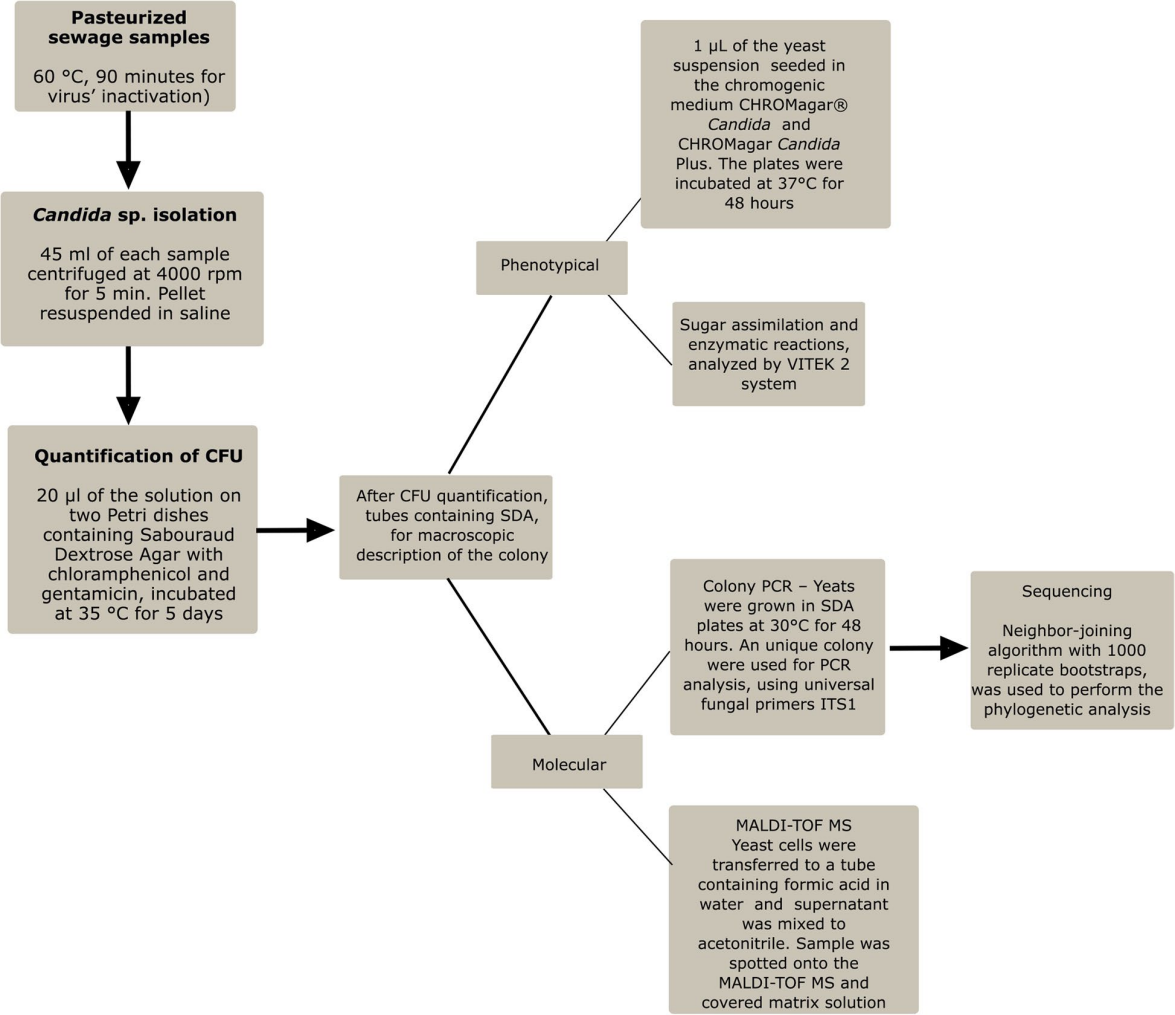
Workflow for yeasts identification by polyphasic taxonomy

In this context, we propose a new identification protocol, using molecular tools (Fig. 4). To obtain the DNA, whose ITS region was sequenced, we used the Colony PCR technique instead of conventional PCR. Colony PCR is a powerful tool for quick and easy screening of colonies grown on selective media. It is a strategy to distinguish true positives from false positives, low cost and fast, as the DNA is obtained from colonies grown within 48 h, without the need to purchase commercial extraction kits or to prepare them in-house. In some cases, it is a superior alternative to the old strategy of growing small cultures of multiple colonies, which requires extracting DNA from each culture [55].

Concomitantly to molecular approaches, our group preconizes the use of mass spectrometry attached to a matrix-assisted laser desorption-ionization (MALDI-TOF), as a tool that has been exploited due to its capacity to identify fungal species belonging to different fungal genera [56–58]. This methodology allows accurate identification of the yeasts since several studies of characterization of multiple *Candida* complexes and Saccharomycotina fungi have been compiled [54, 59].

Pathogenic and opportunistic fungi possess an arsenal of virulence attributes that allow them to survive and cause infection in the hostile environment of the human body. These factors interfere with antigen presentation and skew the T-cell response toward a nonprotective Th2 phenotype [60]. The ability to produce different classes of hydrolytic enzymes and to form biofilm are well-known virulence factors involved in *Candida* sp. infections, including *C. albicans* and many non-albicans *Candida* species [61].

In the present work, *C. palmioleophila*, *C. albicans*, *C. krusei*, and *C. utilis* isolates produced moderated Aspartic Protease and Calcium Phytate activity. In addition, 83,33% (10/12) of the isolates were good producers of Esterase. However, none of the isolates were capable of Mroczyńska and Brillowska-Dąbrowska [62] also evaluated the production of hydrolytic enzyme isolates of *C. palmioleophila* and demonstrated that all of them (100%) produced aspartic protease activity, and, unlike us, two (66.7%) isolates produced phospholipase activity. Pandey et al. [63] evaluated the activities of extracellular hydrolytic enzymes from different *Candida* spp. and observed that *C. krusei* failed to produce the esterase enzyme, corroborating what we observed in this species. However, 54.16% of *C. tropicalis* were strong esterase producers, contrary to what we observed. Regarding phospholipase production, the authors describe *C. krusei* and *C. tropicalis* as strong producers, while in our study, these species were unable to produce this enzyme.

When evaluating biofilm formation, it was possible to observe that all isolates could form a biofilm, with two isolates of *C. palmioleophila* having the highest means of absorbance for the biomass produced. Our results corroborate what was observed by Mroczyńska and Brillowska-Dąbrowska [62] who reported the biofilm formation capability of the three *C. palmioleophila* isolates included in their study. The third largest mean of absorbance was presented by *C. tropicalis*, described as a greater biofilm producer in the *Candida* genus [64], whose mature biofilms are composed of a dense network of blastoconidia and a large amount of extracellular matrix composed of carbohydrates, proteins, phosphorus, uronic acid, and hexosamine [65]. This ability confers various advantages to the microorganisms, including resistance to external aggressors, such as host immune responses and antifungal agents [61].

To sum up, our study points out the impact of global changes and the COVID-19 pandemic on the increasing number of new emerging fungal pathogens that need careful identification, due to modifications in their virulence profiles, which increase the incidence, lethality, and morbidity of fungal diseases. In this sense, we propose a workflow for screening wastewater samples and the inclusion of a new methodology in the identification protocol, applying highly effective molecular tools to discriminate species, using colony PCR, a simple, fast, and inexpensive technique, instead of conventional PCR. We emphasize that the One Health approach applied in our study will enable the early detection of emerging pathogenic yeasts, enabling rapid and accurate diagnosis, necessary to assist in choosing the best therapeutic regimen for the patient, paying attention to antifungal resistance, and culminating in the implementation of strategies for predicting new outbreaks of *Candida* sp.

## Data Availability

Data is provided within the manuscript or supplementary information files.
